# Hepatitis A virus seroprevalence in South Africa - Estimates using routine laboratory data, 2005–2015

**DOI:** 10.1371/journal.pone.0216033

**Published:** 2019-06-26

**Authors:** Ahmad Haeri Mazanderani, Nkengafac Villyen Motaze, Kerrigan McCarthy, Melinda Suchard, Nicolette Marie du Plessis

**Affiliations:** 1 Centre for HIV & STIs, National Institute for Communicable Diseases, National Health Laboratory Service, Johannesburg, South Africa; 2 Department of Medical Virology, Faculty of Health Sciences, University of Pretoria, Pretoria, South Africa; 3 Centre for Vaccines and Immunology, National Institute for Communicable Diseases, National Health Laboratory Service, Johannesburg, South Africa; 4 Department of Global Health, Stellenbosch University, Cape Town, South Africa; 5 Outbreak Response Unit, National Institute for Communicable Diseases, National Health Laboratory Service, Johannesburg, South Africa; 6 Chemical Pathology, School of Pathology, Faculty of Health Sciences, University of the Witwatersrand, Johannesburg, South Africa; 7 Department of Paediatrics, Kalafong Provincial Tertiary Hospital, Faculty of Health Sciences, University of Pretoria, Pretoria, South Africa; Centers for Disease Control and Prevention, UNITED STATES

## Abstract

**Introduction:**

South Africa is considered highly endemic for hepatitis A virus (HAV) although few seroprevalence studies have been conducted over the past two decades. The World Health Organization recommends integrating HAV vaccination into national childhood immunization schedules where there is transition from high to intermediate endemicity. As a means of gauging age-specific rates of infection, we report HAV seroprevalence rates among specimens tested for HAV serology within South Africa’s public health sector from 2005–2015.

**Materials and methods:**

Hepatitis A serology results (Anti-HAV IgM, IgG and total antibody) from 2005–2015 were extracted from South Africa’s National Health Laboratory Service’s Corporate Data Warehouse (NHLS CDW), the central data repository of all laboratory test-sets within the public health sector. Results were extracted according to test-set, result, date of testing, health facility, name, surname, age, and sex. Anti-HAV IgG results were merged with total antibody results to reflect anti-HAV seroprevalence. Testing volume, positivity rates and age-specific anti-HAV seroprevalence rates by year and geographic distribution are described.

**Results and discussion:**

A total of 501 083 HAV IgM results were retrieved, of which 16 423 (3.3%) were positive, 484 259 (96.6%) negative and 401 (0.1%) equivocal; and 34 710 HAV total antibody/IgG tests of which 30 675 (88.4%) were positive, 4 020 (11.6%) negative and 15 equivocal. Whereas IgM positivity was highest among the 1–4 year age group (33.5%) and lowest among patients >45 years (<0.5%), total antibody positivity ranged from its lowest level of 52.7% in the 1–4 year age group increasing to levels of >90% only after 25 years of age.

**Conclusion:**

Anti-HAV total antibody testing within the South African public health sector demonstrates seroprevalence rates reach levels >90% only in adulthood, suggesting South Africa could be in transition from high to intermediate endemicity. Prospective studies with geographically representative sampling are required to confirm these findings and evaluate provincial and urban/rural heterogeneity.

## Introduction

Hepatitis A viral (HAV) infection is the leading cause of viral hepatitis globally [1]. It is usually transmitted via the faecal/oral route through ingestion of contaminated food or water. Whereas exposure to HAV during early childhood is predominantly associated with asymptomatic infection, older children and adults often experience symptomatic disease; with increasing severity associated with increasing age.(1) Unlike hepatitis B or C viral infection, HAV disease is usually self-limiting and not associated with chronicity, it is nevertheless associated with considerable economic burden [2].

Anti-HAV antibodies usually appear a few weeks after infection, with both anti-HAV IgM and IgG often present at the time of initial clinical presentation. Whereas IgM levels decline over 3–6 months following infection, IgG usually persists conveying lifelong immunity. Either anti-HAV total antibody or anti-HAV IgG testing can be performed to determine immunity to hepatitis A infection, which occurs as a result of either natural infection or vaccination [3]. Hence, anti-HAV seroprevalence (presence of anti-HAV total antibody or anti-HAV IgG) gives a measurement of susceptibility to new HAV infections and is a useful epidemiological tool to investigate risk amongst different age groups within a population [4].

The World Health Organization (WHO) defines hepatitis A endemicity by anti-HAV age-seroprevalence for a population. High endemicity being a seroprevalence of ≥90% by 10 years of age, intermediate if seroprevalence is <90% by 10 years but ≥50% by 15 years of age, low if seroprevalence is <50% by 15 years but ≥50% by 30 years of age, and very low if seroprevalence is <50% by 30 years of age [4]. In highly endemic countries large-scale vaccination programmes are deemed unnecessary as almost all persons are asymptomatically infected with HAV during early childhood; thereby effectively preventing clinical hepatitis A in later life [4]. With a change from high to intermediate endemicity, however, the incidence of clinically apparent hepatitis A increases, with countries in transition demonstrating HAV infection as a leading cause of fulminant hepatic failure [4]. On account of this the WHO recommends HAV vaccination be integrated into national immunization schedules for children in populations where seroprevalence drops to <90% by 10 years of age [4]. In South Africa, HAV vaccination is recommended within the private health sector, comprising part of the routine childhood immunisation schedule. However, within the public health sector HAV vaccination is currently not part of the expanded programme on immunization. Hence, the presence of anti-HAV total antibodies or IgG among individuals attending public health facilities is likely the result of natural infection. If national seroprevalence patterns are found to reflect intermediate endemicity across the general population, implementation of a national HAV vaccination policy may be considered.

Several factors have been found to contribute to hepatitis A seroprevalence. In particular, a rise in socioeconomic indicators, such as income, and an increase in access to clean water strongly correlate with a decreased incidence of HAV infection and therefore lower age-seroprevalence rates, especially among children [5,6]. Southern Africa is considered to have high endemicity for hepatitis A with seroprevalence among children aged 1–4 years and 5–9 years estimated to be 83% and 92% respectively [7]. Furthermore, it is thought that the region has remained highly endemic for HAV for the years 1990 to 2005 [7]. Although the true burden of HAV disease is currently unknown, previous serological studies from South Africa have demonstrated that variables associated with higher-socioeconomic status were significantly associated with increased susceptibility to symptomatic HAV infection [8–10]. Whereas South Africa has undergone significant social transformation over the past two decades, with 92.5% of households now having access to clean drinking water and adequate sanitation facilities, increasing from 62.3% in 2002 [11], it remains to be determined whether South Africa has transitioned to a level of intermediate HAV endemicity. We report the HAV seroprevalence rates among specimens submitted for HAV testing between 2005 and 2015 within South Africa’s public health sector.

## Materials and methods

This was a cross-sectional descriptive study of routine laboratory data from the South African public health sector from 2005 to 2015. All anti-HAV serology results (IgM, IgG and total antibody) were extracted from the National Health Laboratory Service’s Corporate Data Warehouse (NHLS CDW), the central data repository of laboratory results within the public health sector in South Africa. Results were extracted according to test-set, qualitative result, date of testing, health facility, and patient demographic details, including name, surname, age, and sex. Each test episode was assigned a unique numerical identifier generated from the patient demographic details after which patient name and surname were deleted prior to analysis.

Criteria for inclusion in the study were results for anti-HAV IgM, anti-HAV IgG and anti-HAV total antibody that were requested within the NHLS between 01 January 2005 to 31 December 2015. Test-sets with a verified positive, equivocal and negative result were included in the analysis (equivocal results refer to valid results that are neither positive nor negative, and are interpreted as such according to the assay manufacturer’s package insert). Test-sets registered without a qualitative result, tests registered for occupational health and safety or study purposes, and duplicate records were excluded from the analysis.

Data were extracted from the NHLS CDW as a Microsoft Excel spreadsheet, and imported into Stata 14 (StataCorp, Texas, USA) for descriptive analysis. All anti-HAV IgG results were merged with anti-HAV total antibody results to reflect HAV exposure (natural- or vaccine-derived) in patients. As testing to determine HAV immunity was predominantly performed using total anti-HAV antibody assays, we have referred to both of these tests as total antibody testing. To determine acute HAV infection, anti-HAV IgM assays are routinely used within the NHLS.

Anti-HAV IgM and total antibody testing patterns and positivity rates were calculated by age group and province. Positivity rates were calculated as the proportion of positive results over total tested. Results were described per year of testing as well as aggregated for all tests resulted between 2005 and 2015. The annual number of acute HAV cases diagnosed per 100 000 population was calculated per province and nationally, rounded-off to the nearest whole number, using population data from Statistics South Africa.

Permission to access and analyse the data was obtained from the NHLS and ethical clearance was obtained from the Human Research Ethics Committee of the University of the Witwatersrand (M160667).

## Results

From 1 January 2005 to 31 December 2015 a total 523 393 specimens registered for HAV serology were retrieved from the NHLS data warehouse. Of the 520 847 registered specimens that met the inclusion criteria, 501 083 were anti-HAV IgM results (459 992 with a registered age or date of birth) and 34 710 anti-HAV total antibody results (31 160 with a registered age or date of birth). A total of 14 946 specimens had both anti-HAV IgM and total antibody test results.

### Laboratory confirmed acute hepatitis A

Among anti-HAV IgM results, 16 423 (3.3%) were positive, 484 259 (96.6%) were negative, and 401 (0.1%) were equivocal. Among patients with a positive result whose sex was registered within the laboratory information system, 53.0% were male and 47.0% were female (sex was unknown in 1.4% of cases), with 4% of males testing positive compared with 2.7% of females. Two thirds of all laboratory confirmed acute hepatitis A cases (anti-HAV IgM positive) occurred among children <15 years of age, with 25% of laboratory confirmed infections diagnosed among children 1–4 years of age. Excluding infants <1 year of age, who acquire transient immunity due to the passive transfer of maternal antibodies in-utero, older age was associated with a decline in the positivity rate, from its highest level of 33.9% in the 1–4 years age group to levels of ≤0.5% in the ≥40 year age groups, with an overall positivity rate of 3.3% among all specimens tested. Laboratory confirmed acute HAV cases with a documented age / DOB, and testing patterns per age range are shown in Table 1.

**Table 1:** Laboratory confirmed acute Hepatitis A cases (with a documented age / DOB) and testing patterns per year-age range, 2005–2015.

Age range (years)	Equivocal results (%, n/N)	Negative results (%, n/N)	Positive results (%, n/N)	Total results (%, n/N)
**<1**	7 (1.8%)	9 828 (2.2%)	115 (0.8%)	9 950 (2.2%)
**1–4**	70 (17.9%)	7 423 (1.7%)	3 838 (25.0%)	11 331 (2.5%)
**5–9**	49 (12.5%)	8 280 (1.9%)	3 654 (23.8%)	11 983 (2.6%)
**10–14**	32 (8.2%)	10 118 (2.3%)	2 265 (14.8%)	12 415 (2.7%)
**15–19**	39 (10.0%)	21 909 (4.9%)	1 531 (10.0%)	23 479 (5.1%)
**20–24**	31 (7.9%)	42 501 (9.6%)	1 389 (9.1%)	43 921 (9.6%)
**25–29**	25 (6.4%)	59 774 (13.5%)	890 (5.8%)	60 689 (13.2%)
**30–39**	46 (11.7%)	115 896 (26.1%)	886 (5.8%)	116 828 (25.4%)
**40–49**	35 (8.9%)	77 030 (17.3%)	412 (2.7%)	77 477 (16.8%)
**50–59**	23 (5.9%)	49 110 (11.1%)	197 (1.3%)	49 330 (10.7%)
**60–69**	20 (5.1%)	26 538 (6.0%)	100 (0.7%)	26 658 (5.8%)
**70–79**	12 (3.1%)	11 719 (2.6%)	52 (0.3%)	11 783 (2.6%)
**≥80**	3 (0.8%)	4 120 (0.9%)	25 (0.2%)	4 148 (0.9%)
**Total (%, n/N)**	**392 (0.1%)**	**444 246 (96.6%)**	**15 354 (3.3%)**	**459 992 (100.0%)**

**Fig 1:**
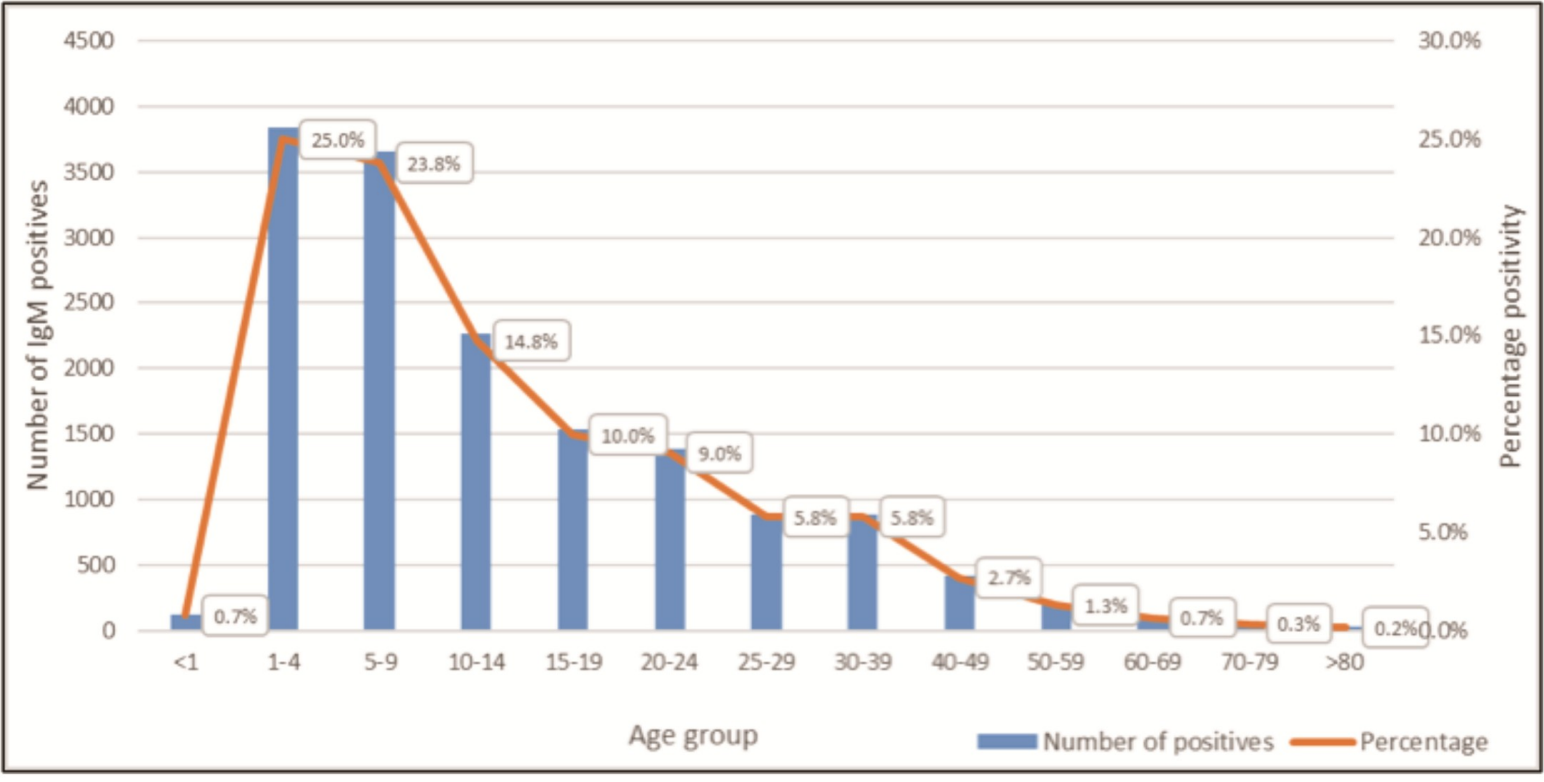
The proportion of laboratory confirmed acute Hepatitis A infections of anti-HAV IgM tests requested per age group, 2005–2015.

**Fig 2:**
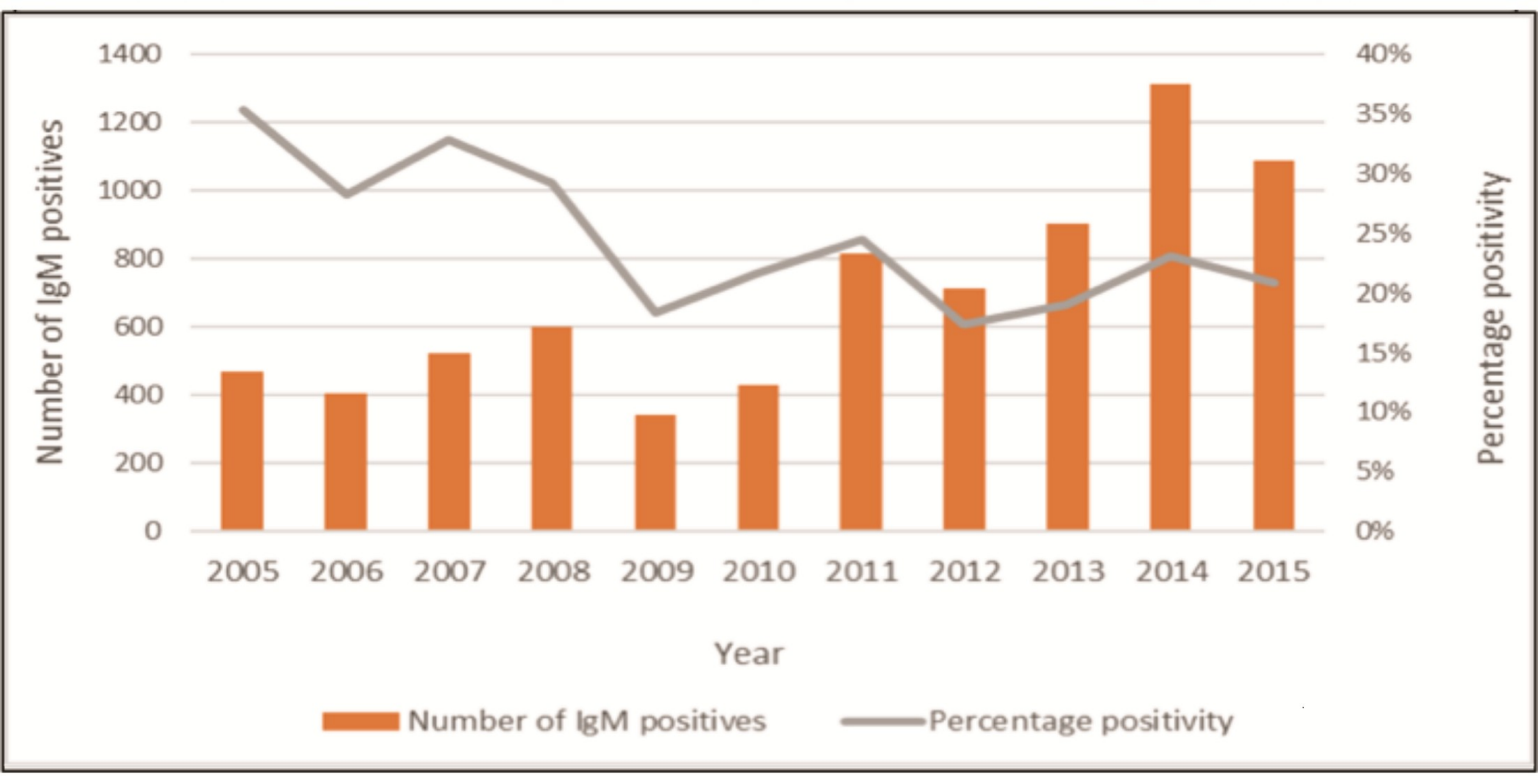
Laboratory confirmed acute Hepatitis A infection among children aged <10 years, 2005–2015.

Between 2005 and 2015, the year with the least number of IgM positive results was 2006, with 715 patients diagnosed with acute hepatitis A, and the year with the greatest number was 2014, with 2 779 patients diagnosed. However, despite the increase in the number of confirmed hepatitis A cases, the positivity rate declined from 8.4% to 2.5% associated with an increase in the annual volume of testing during this period, from 10 096 anti-HAV IgM tests in 2005 to 89 297 in 2015. The 30–39 year age group had the largest IgM testing volume (116 828 results) with a positivity rate of 0.8% (Fig 1). Whereas only 15% of IgM testing was performed among patients <20 years of age, 75% of all positive results occurred in this age group. Restricting analysis to patients <10 years of age, which accounted for 50% of all laboratory confirmed acute hepatitis A cases in the country, an overall decrease in the positivity rate can be appreciated despite increases in the total number of children diagnosed with acute HAV annually (Fig 2).

Considerable variation in anti-HAV IgM testing volumes and positivity rates were observed among South Africa’s nine provinces over the 11-year period for which data was analysed. Among the 500 948 results registered by province, KwaZulu-Natal (187 171) followed by Gauteng (107 446) and the Western Cape (56 952) had the highest number of anti-HAV IgM tests, with the Western Cape having the greatest number of positive results and highest positivity rate (4 217 and 7.4%, respectively), followed by 4 147 (3.9%) in Gauteng and 3 947 (2.1%) in KwaZulu-Natal (Table 2). These three provinces accounted for 75% of all laboratory diagnosed acute hepatitis A cases in the country.

**Table 2:** Laboratory confirmed acute Hepatitis A cases by province 2005–2015.

Province	Equivocal results (%, n/N)	Negative results (%, n/N)	Positive results (%, n/N)	Total results (%, n/N)
**Eastern Cape**	43 (10.7%)	54 202 (11.2%)	895 (5.5%)	55 140 (11.0%)
**Free State**	19 (4.7%)	25 658 (5.3%)	1 149 (7.0%)	26 826 (5.4%)
**Gauteng**	120 (29.9%)	103 179 (21.3%)	4 147 (25.3%)	107 446 (21.5%)
**KwaZulu-Natal**	1 (0.3%)	183 223(37.8%)	3 947 (24.0%)	187 171 (37.4%)
**Limpopo**	4 (1.0%)	23 608 (4.9%)	858 (5.2%	24 470 (4.9%)
**Mpumalanga**	2 (0.5%)	21 037 (4.3%)	683 (4.2%)	21 722 (4.3%)
**North West**	22 (5.5%)	18 235 (3.8%)	436 (2.7%)	18 693 (3.7%)
**Northern Cape**	2 (0.5%)	2 442 (0.5%)	84 (0.5%)	2 528 (0.5%)
**Western Cape**	188 (46.9%)	52 547 (10.9%)	4 217 (25.7%)	56 952 (11.37%)
**Grand Total**	**401 (0.1%)**	**484 131 (96.6%)**	**16 416 (3.3%)**	**500 948 (100%)**

In 2014, the year with the highest number of HAV cases diagnosed equivalent to 5 cases per 100 000 of the national population, KwaZulu-Natal had an estimated 10 cases, Western Cape 9 cases, Free-State, Limpopo, Mpumalanga, North-West and Northern Cape provinces each had 3 cases, and Gauteng 2 cases per 100 000 population.

### Hepatitis A immunity

**Fig 3:**
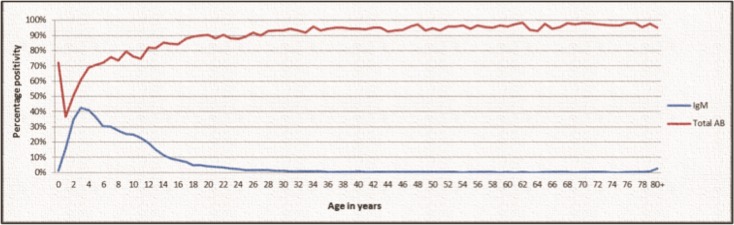
Hepatitis A age-seroprevalence among patients tested within the South African public sector, 2005–2015. IgM, anti-Hepatitis A IgM antibody positivity; Total AB, anti-Hepatitis A total antibody positivity.

Of the specimens tested for anti-HAV total antibody, 30 675 (88.4%) were positive, 4 020 (11.6%) negative and 15 equivocal. Test episodes with an anti-HAV total antibody negative result and a documented sex showed 54.1% were male and 45.9% were female (0.9% of cases sex was unknown). Excluding infants <1 year of age, older age was associated with an increase in seropositivity, with the positivity rate ranging from its lowest level of 52.7% in the 1–4 year age group to levels >90% in the >25 year age groups. Anti-HAV seropositive cases and rates among different age groups for the study period are illustrated in Table 3. Fig 3 represents age as a continuous variable and allows visualization of HAV seroprevalence alongside the acute hepatitis A positivity rate. A seropositive rate of 90% was reached only well after 10 years of age.

**Table 3:** Hepatitis A seropositive cases (%) by age-range and year, 2005–2015.

Year	Age (years)													
	<1	1–4	5–9	10–14	15–19	20–24	25–29	30–39	40–49	50–59	60–69	70–79	≥80	Total
**2005**	24 (66.7%)	71 (47.7%)	62 (65.3%)	84 (70.6%)	33 (66.0%)	32 (71.1%)	31 (59.6%)	81 (81.0%)	68 (85.0%)	40 (85.1%)	18 (94.7%)	8 (88.9%)	2 (66.7%)	**554 (68.9%)**
**2006**	27 (50.9%)	63 (44.4%)	65 (61.9%)	77 (75.5%)	53 (80.3%)	30 (73.2%)	33 (70.2%)	56 (83.6%)	60 (81.1%)	41 (85.4%)	20 (100%)	9 (100%)	2 (100%)	**536 (69.1%)**
**2007**	16 (45.7%)	79 (47.3%)	94 (62.7%)	99 (80.5%)	54 (80.6%)	71 (76.3%)	78 (83.9%)	116 (88.6%)	99 (90.8%)	58 (84.1%)	33 (100%)	15 (93.8%)	3 (100%)	**815 (74.8%)**
**2008**	42 (59.2%)	92 (51.1%)	113 (67.3%)	128 (80.5%)	102 (85.0%)	125 (84.5%)	150 (92.6%)	280 (90.6%)	220 (93.6%)	134 (97.8%)	82 (94.3%)	33 (94.3%)	11 (84.6%)	**1 512 (82.9%)**
**2009**	44 (56.4%)	96 (47.3%)	125 (74.0%)	129 (77.7%)	123 (88.5%)	147 (86.5%)	200 (85.8%)	378 (90.0%)	278 (94.2%)	190 (94.1%)	99 (95.2%)	35 (94.6%)	13 (100%)	**1 857 (83.3%)**
**2010**	81 (72.3%)	114 (54.8%)	144 (73.9%)	131 (78.0%)	179 (89.5%)	272 (88.9%)	424 (92.8%)	736 (94.5%)	470 (95.5%)	304 (94.1%)	135 (93.8%)	73 (100%)	14 (100%)	**3 077 (88.7%)**
**2011**	310 (85.4%)	297 (61.9%)	349 (80.1%)	355 (85.5%)	422 (92.8%)	886 (93.7%)	1 487 (95.7%)	2 719 (97.3%)	1 581 (97.2%)	943 (97.3%)	472 (98.1%)	175 (99.4%)	61 (98.4%)	**10 057 (93.5%)**
**2012**	65 (71.4%)	120 (53.1%)	156 (81.7%)	133 (77.8%)	180 (88.2%)	251 (89.6%)	395 (93.6%)	709 (95.8%)	441 (95.0%)	289 (97.0%)	172 (97.2%)	88 (95.7%)	26 (100%)	**3 025 (89.4%)**
**2013**	33 (67.4%)	91 (45.3%)	113 (72.0%)	114 (82.0%)	118 (90.1%)	166 (86.9%)	242 (86.1%)	418 (87.1%)	327 (89.1%)	262 (94.9%)	120 (96.0%)	64 (97.0%)	26 (96.3%)	**2 094 (84.1%)**
**2014**	43 (68.2%)	101 (51.8%)	140 (75.3%)	117 (77.5%)	107 (78.7%)	163 (86.2%)	230 (85.8%)	457 (91.2%)	336 (93.1%)	288 (95.4%)	170 (92.9%)	91 (96.8%)	33 (100%)	**2 276** **(85.5%)**
**2015**	24 (75.0%)	63 (63.6%)	92 (87.6%)	58 (80.6%)	66 (83.5%)	116 (87.2%)	174 (90.6%)	343 (93.2%)	247 (92.9%)	182 (94.8%)	96 (94.1%)	38 (97.4%)	14 (93.3%)	**1 513 (89.3%)**
**Total**	**709** **(72.1%)**	**1 187** **(52.7%)**	**1 453 (74.3%)**	**1 425 (79.8%)**	**1 437** **(87.3%)**	**2 259** **(88.9%)**	**3 444 (91.6%)**	**6 293 (94.1%)**	**4 127 (94.4%)**	**2 731 (95.4%)**	**1 417 (96.1%)**	**629 (97.4%)**	**205 (97.2%)**	**27 316 (87.6%)**
*Seropositivity (%) within age-range*				**≥90%**			**≥75-<90%**			**≥50-<75%**			**<50%**	

Both anti-HAV total antibody and anti-HAV IgM tests were requested in 14 946 patients during the study period. Cross tabulation of the results, as illustrated in Table 4, show that 643 (4.3%) of those tested for anti-HAV total antibodies had an acute HAV infection.

**Table 4:** Cross tabulation of test-sets where both anti-HAV total antibody and anti-HAV IgM tests were requested, 2005–2015.

Anti-HAV IgM antibodies	Anti-HAV total antibodies			
	Negative	Positive	Equivocal	Total
Negative	1 039	13 230	2	**14 271**
Positive	33	614	0	**647**
Equivocal	0	28	0	**28**
**Total**	**1 072**	**13 872**	**2**	**14 946**

The volume of anti-HAV total antibody tests increased from 804 in 2005, peaking at 10 758 tests in 2011, and thereafter declining to 1 694 in 2015. Both the number and proportion of positive total antibody results increased with the number of tests performed, ranging from 554 (68.8%) in 2005, to a peak of 10 057 (93.5%) in 2011, and thereafter declining to 1 513 (89.3%) in 2015 (Table 3).

Provincial variation in anti-HAV total antibody testing were also observed, with the majority of tests performed in the Western Cape, KwaZulu-Natal and Gauteng (Table 5). Among these three provinces anti-HAV total antibody seropositivity was highest in KwaZulu-Natal (95.8%), then Western Cape (86.1%) and Gauteng (64.5%) (Table 4).

**Table 5:** Hepatitis A seropositive cases (%) by province 2005–2015.

Province	Equivocal results (%, n/N)	Negative results (%, n/N)	Positive results (%, n/N)	Total results (%, n/N)
**Eastern Cape**	2 (13.3%)	88 (2.2%)	2 126 (6.9%)	2 216 (6.4%)
**Free State**	2 (13.3%)	96 (2.4%)	486 (1.6%)	584 (1.7%)
**Gauteng**	0 (0.0%)	1 000 (24.9%)	1 825 (6%)	2 825 (8.1%)
**KwaZulu-Natal**	0 (0.0%)	567 (14.1%)	13 232 (43.2%)	13 799 (39.8%)
**Limpopo**	0 (0.0%)	54 (1.4%)	36 (0.1%)	90 (0.3%)
**Mpumalanga**	0 (0.0%)	38 (0.9%)	6 (0%)	44 (0.1%)
**North West**	0 (0.0%)	87 (2.2%)	52 (0.2%)	139 (0.4%)
**Northern Cape**	0 (0.0%)	18 (0.4%)	68 (0.2%)	86 (0.3%)
**Western Cape**	11 (73.4%)	2 070 (51.5%)	12 827 (41.8%)	14 908 (42.9%)
**Grand Total**	**15 (0.04%)**	**4 018 (11.60%)**	**30 658 (88.36%)**	**34 691 (100%)**

## Discussion

This study represents the largest description of HAV seroprevalence within South Africa to date. Using routine laboratory data, hepatitis A testing patterns, acute infection and seropositivity rates within the public health sector are described and the level of endemicity gauged.

Although previous studies from southern Africa have described the region as highly endemic for HAV, routine laboratory data presented here suggest South Africa has a level of intermediate endemicity with anti-HAV seroprevalence reaching a level of >90% only among adults >25 years of age. These findings are in keeping with the last published serological survey conducted in South Africa which reported seropositivity of 80% among children 11–13 years of age from low socio-economic groups (and a much lower seroprevalence among children from affluent backgrounds) [10]. Unfortunately, we were unable to accurately determine yearly seroprevalence trends, despite data obtained from 2005 to 2015, on account of the relative low volume of anti-HAV total antibody testing and uneven distribution of testing among the various age groups. Nevertheless, aggregated data over the 11-year period portrays a compelling picture whereby total antibody seroprevalence and IgM positivity follow opposite trajectories during childhood, both of which plateau at around 25 years of age, reflecting minimal infection once immunity reaches levels of >90%. Although the volume of anti-HAV IgM testing performed among adults was found to increase over the years, and was associated with a marked reduction in percentage positivity, the number of laboratory confirmed acute hepatitis A cases has increased considerably each year, by a factor of three, over the past decade. Whereas 1 612 cases of Hepatitis A were notified between the years 2001 and 2005, more cases were diagnosed in 2015 alone highlighting the need for better electronic notification systems as well as raising concerns regarding the potential increase in symptomatic acute infection.

Symptomatic illness from HAV infection is directly related to age, with 70% of pre-school children having no symptoms whereas >70% of infected adults present with jaundice [12]. Hence, HAV infection among young children is expected to be under-reported. Yet despite this, routine laboratory data suggests that children 1–4 years of age comprised the highest proportion (33.5%) of acute hepatitis A cases compared to all other age groups. Furthermore, the data suggest that in contrast to children, adults with symptoms of hepatitis are unlikely to have hepatitis A infection within the South African context, with <0.5% of those aged >45 years testing positive yet comprising a quarter of all IgM tests performed. We suggest that commonly used algorithms for serological diagnosis of viral hepatitis should be reviewed to ensure age-appropriate hepatitis serology testing which in turn will lead to appropriate and cost-effective laboratory utilization [13–14]. Hence, clinical requests for ‘viral hepatitis testing’ should not routinely include HAV IgM testing for all patients. These findings lay groundwork for further investigation to determine age-specific seroprevalence rates among healthy individuals, especially children and adolescent age groups, within South Africa.

Inter-provincial variations were seen with both acute hepatitis A cases and seroprevalence. However, on account of variations in access to testing, different clinical testing algorithms and incomplete data within the data warehouse it is not possible to accurately determine geographic differences in incidence of HAV infection using routine laboratory data. The fact that the Western Cape was the province with the highest number and proportion of IgM positive tests and had a relatively low overall anti-HAV seroprevalence rate suggests inter-provincial differences are likely to exist and should be taken into consideration when planning prospective seroprevalence studies.

## Limitations

A number of important limitations exist regarding this study. Data from NHLS CDW was used which reflects testing practices within the South African public health sector only. This analysis may therefore not necessarily reflect the population as a whole as more affluent sectors of South African society would not have been included. Approximately 20% of the South African population engage with the private health sector [15], and this component of the population is likely to have a much lower infection rate as a result of private vaccination practice and better living conditions. When infection does occur within the private sector, it is likely to arise at an older age with a greater proportion of infections associated with symptomatic disease. It is important to note that clinical data, including patient socio-economic status, is not captured within the NHLS CDW thereby precluding a description of these variables and their association with HAV seroprevalence within the South African public health sector.

As testing for anti-HAV IgM is usually performed within the clinical context of acute hepatitis, it can be assumed that the overwhelming majority of patients tested for IgM had presented with clinical disease. Reasons for testing anti-HAV total antibody, however, are much less clear with 60% of anti-HAV total antibody testing performed without simultaneous IgM testing. Hence, seroprevalence findings from routine laboratory data may not be representative of the general population. An additional limitation relates to the assays used, for which data was not extracted. Various different HAV serological assays are used within the NHLS, although it is understood that all HAV serology assays used are approved for *in-vitro* diagnostic use.

Although increase in HAV IgM testing volume likely reflects increased access to healthcare between the years 2005 to 2015, data was incomplete for KwaZulu-Natal province prior to 2010 and therefore does not reflect the total number of test-sets within the public sector.

Lastly, on account of transcription errors, linking of results and de-duplicating results for individual patients using probabilistic matching of demographics has flaws; the patient linking-algorithm used in this study has a reported sensitivity of 73% for patient-linkages with 83% of matches correctly linking test results to the same patient [16].

### Conclusions

Among patients tested within South Africa’s public health sector from 2005 to 2015, acute hepatitis A cases occurred mostly amongst children <15 years of age with few cases occurring among adults despite considerable testing. Anti-HAV total antibody results demonstrate that seroprevalence rates reach levels >90% only during adulthood among individuals aged >25 years, suggesting South Africa is a country of intermediate endemicity. The very low percentage positivity of anti-HAV IgM results among adults aged >35 years suggest that this test is not essential as a first line screening tool for adults with suspected acute hepatitis. Further seroprevalence studies using representative population-based samples are needed to confirm these findings and inform the national vaccination program.

## Supporting information

S1 Dataset (XLSX)
